# Polyethyleneimine-Functionalized Carbon Nanotubes Enabling Potent Antimycotic Activity of Lyticase

**DOI:** 10.3390/polym14050959

**Published:** 2022-02-28

**Authors:** Weibing Liang, Ming Chen, Lin Li, Liqiang Yan, Xiuli Wang, Xiongzhi Wu, Chenghong Lei

**Affiliations:** College of Chemistry and Bioengineering, Guilin University of Technology, Guilin 541006, China; 1020190482@glut.edu.cn (W.L.); liqiangyan@glut.edu.cn (L.Y.); wangxiuli1109@163.com (X.W.); 2004046@glut.edu.cn (X.W.)

**Keywords:** lyticase, polyethyleneimine, functionalization, carbon nanotubes, antimycotic activity

## Abstract

In this work, the positively-charged polymer polyethyleneimine was used to functionalize carbon nanotubes and activated carbon to load antimycotic enzyme lyticase. Interestingly, polyethyleneimine played a dual role functionalizing carbon materials to synergistically enhance antimycotic activity of loaded lyticase as well as exhibiting its own apparent antimycotic activity, where the enhanced enzymatic activity of loaded lyticase on functionalized carbon nanotubes was more than 2.8 times as high as the activity of free enzyme in solution. The actual activity of loaded lyticase on functionalized carbon nanotubes was applied with *Penicillium janthinellum*, exhibiting much faster digesting lysis of the bacteria in comparison with free lyticase. The synergistic and potent antimycotic activities from combined action of antimycotic lyticase and polyethyleneimine on carbon nanotubes provides a new antimycotic protection for medicine, food industry, and other biochemical processes.

## 1. Introduction

Polyethyleneimine (PEI) is a linear or branched or dendrimeric synthetic polymer with repeating unit composed of the amine groups and two carbon aliphatic CH₂CH₂ spacer. Linear PEI is comprised of secondary amines and branched or dendrimeric PEI and contains primary, secondary, and tertiary amino groups, where all types of PEIs have primary amines as terminal groups [1]. PEI is water-soluble and has been extensively used in many areas due to its polycationic and positively-charged characters, including cell culture [2], gene transfer, transfection and delivery [3,4,5,6,7], CO_2_ capturing [8,9,10], and so on.

Lyticase (LYT, also known as zymolyase) is an enzyme complex exhibiting antimycotic activity and the enzyme complex is preferred to digest cell walls of yeast and generate spheroplasts from fungi for transformation [11,12,13,14]. Essential activities of lyticase include β-1,3-glucan laminaripentao-hydrolase activity and β-1,3-glucanase activity. Yeast cells and fungi are difficult to disrupt because the cell walls may form capsules or resistant spores. Loaded lyticase on solid supporting materials could find many applications in antimycotic protection for medicine, food industry, and other biochemical processes.

The selection of loading materials or immobilizing matrices for enzymes have not been well solved and the specific activity of the loaded enzyme is often lower than that of the free enzyme in solution prior to the immobilization [15,16,17,18]. Considering the particularity of LYT, whose substrate size such as yeast cell is in the range of microns, conventional porous and encapsulation materials are not suitable for carrying LYT. With this in mind, carbon nanotubes (CNT) could be the suitable materials for loading LYT. CNT has been used as immobilizing matrices for some enzymes [19,20,21,22,23]. However, enzymes and proteins often encounter some activity loss when covalently conjugated or spontaneously loaded with carbon materials because of carbon materials’ large surface areas and strong adsorption property leading to harmful polarization of loaded biomolecules. Appropriate functionalization of carbon materials could be the efficient way to alleviate such harmful effects and may enhance and stabilize the activities of loaded biomolecules [24,25].

The positively-charged PEI is widely used due to its polycationic ability to interact with a variety of negatively-charged moieties. PEI has been used for wrapping CNT as electrochemical detection platforms [26,27,28,29], and as a compatibilizer to improve dispersion of CNT [28,30]. It has been reported that the positively-charged polylysine can adsorb electrostatically and, thereby, strip the outer membrane of bacterial cells, causing damage to the bacterial cells [31]. Similarly, PEI could adsorb electrostatically to the negatively-charged surface of yeast cells as well [32]. In this work, especially, we employed the positively-charged PEI to functionalize CNT for carrying and loading the antimycotic enzyme LYT. Importantly, not only PEI-functionalized carbon nanotubes (FCNT: CNT-PEI) displayed their outstanding apparent antimycotic activity, but also as a functionalizing reagent, PEI synergistically enhanced the activity of the loaded LYT on FCNT (CNT-PEI-LYT) resulting in potent antimycotic activities, in sharp contrast to that with activated carbon (AC) or PEI-functionalized activated carbon (FAC: AC-PEI) under the same conditions.

## 2. Materials and Methods

A pH 7.4, phosphate buffered saline (PBS, 10 mM phosphate buffer containing 0.0027 M potassium chloride and 0.137 M sodium chloride) (from Sigma-Aldrich, St. Louis, MO, USA. P4417) was used as the working buffer for this work. Two milligrams of multi-wall carbon nanotubes (CNT) (from Aladdin, Shanghai, China) or activated carbon (AC) (from NaturaLife Labs, Torrance, CA, USA) was added with 0.8 mL of the working buffer and allowed to be sonicated for 10 min, then it was centrifuged at 8000 RPM for 2 min and the supernatant was removed, repeating this process twice. The resulting carbon material was resuspended in 0.4 mL of the working buffer by sonicating for 10 min. The suspension of CNT or AC was mixed and shaken with 0.4 mL of 1.0 mg/mL polyethyleneimine (PEI) (M.W. 10,000, from Aladdin, Shanghai, China) in pH 7.4, PBS at 800 RPM for 2 h. Then it was centrifuged at 8000 RPM for 2 min. Then the supernatant was removed and added with 0.8 mL of the working buffer and allowed to be sonicated for 10 min, then it was centrifuged at 8000 RPM for 2 min and the supernatant was removed, repeating this process twice. The PEI-functionalized carbon material, CNT-PEI or AC-PEI was resuspended in 0.77 mL of the working buffer and allowed it to be sonicated for 10 min for use.

The suspension of CNT-PEI or AC-PEI was added with 30 µL of 1.0 mg/mL lyticase from *Arthrobacter luteus* (LYT, from Sigma-Aldrich, St. Louis, MO, USA. L2524) in the working buffer in a 1.8 mL tube. Then, it was sonicated in an ice bath for 10 min. The suspension was shaken at 800 min^−1^ on a thermomixer at 8–10 °C for 2 h. Then, the enzyme–carbon material suspension was centrifuged at 8000 RPM for 2 min, the supernatant was removed, and the resulting enzyme–carbon composite was washed by 0.7 mL of the working buffer and centrifuged to remove any non-firmly immobilized enzyme, repeating this process twice. Finally, the supernatants were combined together and the washed deposit was resuspended in 0.8 mL of the working buffer for further protein and activity measurement.

In this work, unit definition of LYT is that one unit of lysozyme will produce a ∆A_800_ of 0.001 per minute at pH 7.4, PBS at 25 °C using a suspension of yeast cells from *Saccharomyces cerevisiae* (from Sigma-Aldrich, 51475) as substrate in a 1.5 mL reaction mixture. The 30 µL of the working buffer, the LYT solution or the carbon material or enzyme–carbon material suspension (2 mg of the AC or CNT based material loaded with 0.02–0.05 mg of LYT suspended in 0.8 mL of the working buffer) was added into the 1-cm cuvette containing 495 µL H_2_O, 0.75 mL of the working buffer and 225 µL of 4.0 mg/mL yeast in the working buffer under stirring at 25 °C. It was blanked for enzymatic activity or apparent activity measurement with the same buffer background. The spectrophotometer started to record kinetic change of absorbance at 800 nm as soon as the enzyme or the control example was added. For measuring the actual antimycotic activity on *Penicillium janthinellum*, 2 mL of *Penicillium janthinellum* cell suspension was added with 0.1 mL of the samples of LYT, FCNT, or FCNT-LYT under stirring and the kinetic change of UV absorbance was recorded at 470 nm, where the initial absorbance of the cell suspension was controlled in the range from 0.32 to 0.35.

All protein amounts were measured using a BCA Assay Kit. An *Agilent* 8453 Diode Array Spectrophotometer was used for measurement of enzymatic activity, apparent activity, and protein amount. Fluorescence spectra were measured with a F7000 fluorometer (Hitachi, Tokyo, Japan) for the same samples as for CD measurement. The scanning electron micrograms (SEM) were obtained with a SU5000 scanning electron microscope (Hitachi, Tokyo, Japan) at an accelerator voltage of 5 kV. The transmission electron micrograms (TEM) were taken using JEM-2100F transmission electron microscope with the operating voltage of 200 kV.

## 3. Results and Discussion

**Figure 1:**
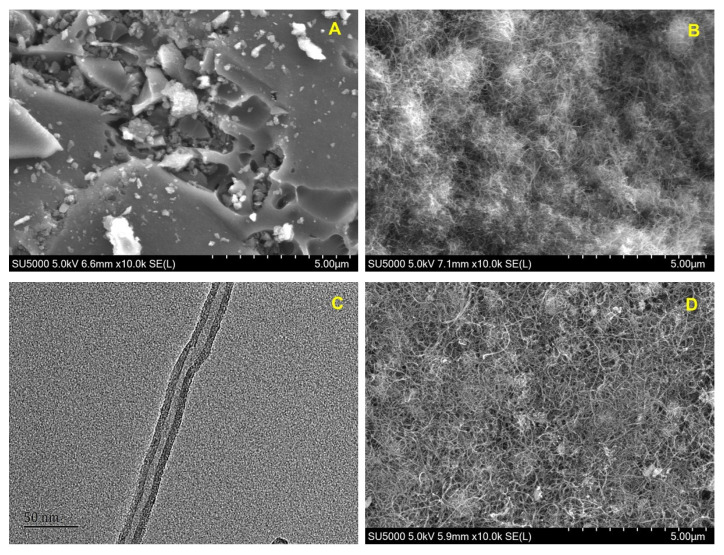

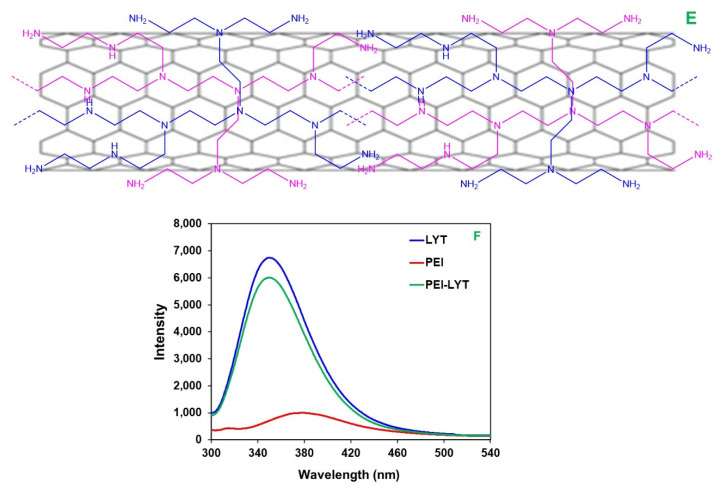
(**A**) SEM images of AC; (**B**) SEM image of CNT; (**C**) TEM images of CNT; (**D**) SEM image of CNT-PEI; (**E**) Schematic draw of PEI-coated CNT (CNT-PEI) with typical branched PEI fragments; (**F**) Fluorescence emission spectra of LYT, PEI, and PEI-LYT. The final concentrations of LYT and PEI in pH 7.4, PBS were 0.0375 mg/mL and 0.5 mg/mL, respectively. Excitation wavelength: 280 nm.

Figure 1 shows the scanning electron microscopy (SEM) images of AC and CNT. The SEM image shows AC has its structure of amorphous and porous carbon block (Figure 1A), while CNT displays the typical structure of fine nanotubes entangled and partially sintered together (Figure 1B). The TEM image of CNT displayed the high-resolution structure of a multi-wall carbon nanotube (Figure 1C). Although PEI can help dispersion of CNT-PEI in solution as reported [28,30], the SEM image of CNT-PEI still shows the CNT agglomeration due to the sample deposition (Figure 1D). Except for the polymer coating and deposition, CNT had no dramatical mechanical change after PEI functionalization (Figure 1D). Figure 1E shows schematic draw of PEI-coated CNT (CNT-PEI) with typical branched PEI fragments.

In this work, PEI was used for alleviating the harmful effects of CNT for loading the enzyme LYT. Therefore, we first studied the interaction of PEI and LYT by fluorescence spectroscopy. Fluorescence emission spectra were used to assess the microenvironmental changes of tyrosinyl (Tyr) and tryptophanyl (Trp) residues in LYT when interacting with PEI. Figure 1F shows fluorescence emission spectra of LYT, PEI, and PEI-LYT. Fluorescence emission was monitored at the excitation wavelength of 280 nm, allowing excitation of both tyrosinyl and tryptophanyl residues. The peak wavelengths are centered at 350 nm for LYT and PEI-LYT indicating that there was no dramatic conformational change of LYT in the presence of PEI (Figure 1F). However, the emission intensity of LYT was somewhat decreased in the presence of PEI (Figure 1F), demonstrating that the interaction of PEI affected some exposure of the related residues. The result indicates that there may be some conformational change resulted from the interaction of LYT with PEI. The later result would show this conformational change were favorable.

The multi-wall CNT or AC was functionalized by immersing in PEI solution, where PEI was spontaneously adsorbed on CNT. The carbon materials were incubated in the enzyme stock solution, thereby avoiding harsh immobilization conditions that destroy enzymatic activity. The enzyme was spontaneously adsorbed on the carbon materials. By increasing the ionic strength and changing the pH value with 0.5 M NaCl and pH 9.4, PBS instead of the working buffer (pH 7.4, PBS), we found there was no enzyme desorption/leaking, indicating that the strong adsorption is not attributed to electrostatic interaction, but a comprehensive interaction. The adsorption of the spontaneously loaded enzyme on functionalized carbon nanotube was stable, indicating that the strong adsorption is not dominated by electrostatic interaction, but a comprehensive interaction. The comprehensive strong interaction may include van der Waals force, H bond, electrostatic, hydrophilic, hydrophobic, and other interactions.

We measured the protein-loading density of the enzyme on CNT and determined the specific (antimycotic) activity of the loaded LYT and the apparent antimycotic activity of the carbon materials in the absence of LYT in comparison with that of the free lysozyme in solution. We define the protein amount (mg) of an enzyme loaded on one milligram of carbon materials (functionalized or unfunctionalized) as the protein loading density (P_LD_), and the corresponding activity (units) as the activity loading density (A_LD_), where A_LD_/P_LD_ is defined as the specific activity of the load enzyme. The activity of LYT was measured based on the absorbance decrease of the suspension of the substrate yeast cells from *Saccharomyces cerevisiae* at 800 nm.

**Figure 2:**
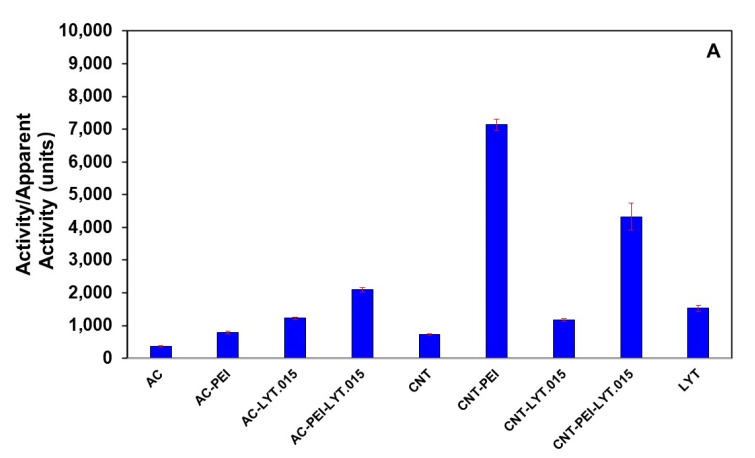

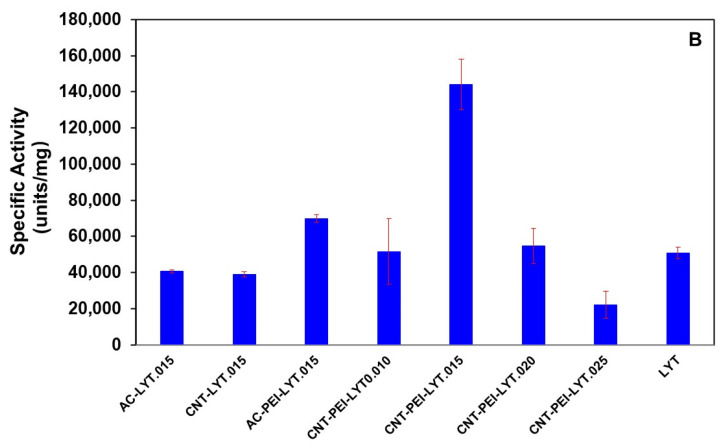
(**A**) Apparent activities of AC, CNT, FAC, or FCNT and general activities of loaded LYT on AC, CNT, FAC, or FCNT when P_LD_ of LYT was 0.015 mg/mg on 2 mg of carbon materials, comparing to general activity of 0.03 mg LYT free in solution; (**B**) Specific activities of loaded LYT on AC, CNT, FAC, or FCNT at different P_LD_ of LYT comparing to specific activity of free LYT in solution. 0.02–0.05 mg of LYT was loaded on 2 mg of carbon materials. LYT.010—LYT.025 represent the loading density of LYT on carbon materials being in the range from 0.010 mg/mg to 0.025 mg/mg. The loading density of PEI was 0.2 mg/mg. Working solution: pH 7.4, PBS. Specific activity of LYT: 50840.53 ± 3098.41 units/mg.

In the absence of LYT, the substrate yeast cells can be adsorbed on both unfunctionalized and functionalized carbon materials leading to the decrease of the absorbance of the cell suspension at 800 nm, thus the apparent activity can be calculated based on the similar method to that in the presence of LYT. The general activity of the loaded LYT was measured by subtracting the apparent activity of unfunctionalized or functionalized carbon materials (Figure 2A), where the loading density of PEI spontaneously adsorbed on unfunctionalized AC or CNT (2 mg) was controlled to be 0.2 mg/mg and P_LD_ of LYT on functionalized or unfunctionalized carbon materials was controlled to be 0.015 mg/mg. Figure 2A shows apparent activities or general activities of a variety of carbon materials in the presence and absence of loaded LYT in comparison with the general activity of free LYT(0.03 mg) in solution. The apparent activities of FAC and FCNT with PEI were much larger than AC and CNT (Figure 2A), respectively, implying that PEI might play an important role in enhancing antimycotic activity of LYT. At P_LD_ of LYT with 0.015 mg/mg on 2 mg of carbon materials, the general activities of loaded LYT on AC and FAC were higher than the apparent activities of AC and FAC, respectively (Figure 2A). The general activity of loaded LYT on CNT was higher than the apparent activity of CNT. However, interestingly, the apparent activity of FCNT (CNT-PEI) was apparently even higher than the general activity of loaded LYT on FCNT. In fact, FCNT indeed exhibited some antimycotic activity on the tested cells of *Penicillium janthinellum*, as discribed later.

The specific activity of loaded LYT on various carbon materials was obtained by dividing the general activity with the loaded protein amount. Figure 2B shows specific activities of free LYT in solution and loaded LYT on a variety of carbon materials, where P_LD_ of LYT on various carbon materials was controlled to be in the range from 0.010 to 0.025 mg/mg. At P_LD_ of LYT with 0.015 mg/mg, the specific activity of loaded LYT on AC and CNT was decreased in comparison with the specific activity of free LYT, while the specific activity of loaded LYT on FAC was increased by 37% (Figure 2B). More significant increase of the specific activity of LYT occurred when loaded on FCNT. Dramatically, at P_LD_ of LYT with 0.015 mg/mg, the specific activity of loaded LYT on FCNT (CNT-PEI-LYT.015) was increased to more than 2.8 times as high as that of free LYT in solution (Figure 2B), indicating functionalization of CNT with PEI synergistically increase the specific activity of loaded LYT. The increased specific activities of CNT-PEI-LYT demonstrated the conformational changes change resulted from the interaction of LYT with PEI were favorable (Figure 1F) and thus synergistically enhanced specific activity of LYT. Moreover, P_LD_ of LYT also affected the specific activity when loading on FCNT. Among the different loading densities in the range from 0.010 to 0.025 mg/mg, when P_LD_ of LYT was 0.015 mg/mg, CNT-PEI-LYT displayed the highest activity.

**Figure 3:**
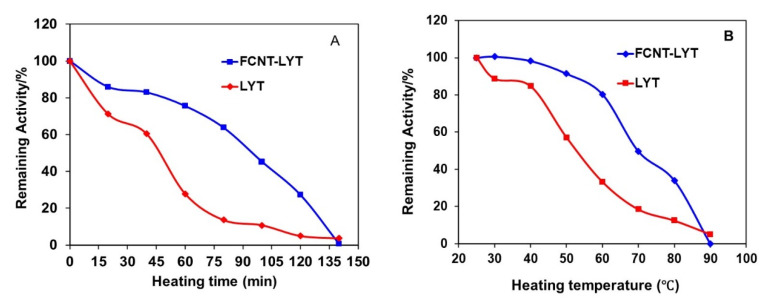
(**A**) Decay of the activity of free LYT and FCNT-LYT (CNT-PEI-LYT) in the working buffer incubated at 60 °C for different time; (**B**) Decay of the activity of free LYT and FCNT-LYT in the working buffer incubated at different temperature for 1 h. P_LD_ of LYT: 0.015 mg/mg. Working buffer: pH 7.4, PBS.

To study whether the enzyme was thermodynamically more stable than the free enzyme in solution, the kinetics of thermal inactivation of the enzyme and FCNT-enzyme were measured. The free LYT lost its activity all the way down to less than 30% of its initial value after incubation at 60 °C for 1 h (Figure 3A). In contrast, the thermal stability of FCNT-LYT with PEI (CNT-PEI-LYT) was dramatically increased. In fact, after incubating FCNT-LYT at 60 °C for 1 h, there was still more than 75% of its initial activity. The half-life time (t_1/2_) of the specific activity for free LYT and FCNT-LYT were 41.15 min and 91.55 min (Figure 3A), respectively, demonstrating that FCNT-LYT had a more than two-fold improvement over free LYT against thermal inactivation. The thermal stability of free LYT and FCNT-LYT as a function of the incubation temperature for 1 h is depicted in Figure 3B, where we define ‘T_t_’ as a transition temperature at which the LYT sample still kept 50% of its initial activity. The results show that T_t_ shifted from 53.3 °C for free LYT to 69.9 °C for FCNT-LYT (Figure 3B), demonstrating a dramatic enhancement of thermal stability upon LYT loading in FCNT.

**Figure 4:**
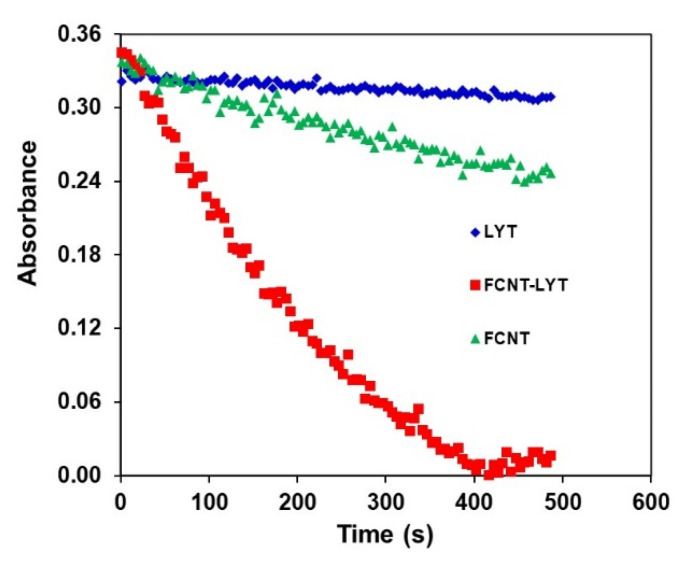
Kinetic change of UV absorbance for measuring antimycotic activity of LYZ and FCNT-LYT with PEI against *Penicillium janthinellum.* The enzyme samples contained 0.0375 mg/mL of LYZ or 2.5 mg/mL of CNT in the working buffer, while the loading density of PEI on CNT was 0.20 mg/mg. The UV absorbance was recorded at 470 nm.

*Penicillium janthinellum* was chosen for testing and comparing the actual antimycotic activity of LYT, FCNT, and FCNT-LYT (CNT-PEI-LYT). The *Penicillium janthinellum* cell suspension in water has a special UV absorption peak at 470 nm. Upon addition of LYT, FCNT, and FCNT-LYT, the absorbance of the bacterial cell suspension was decreased along with the enzymatic digesting of the bacterial cells. FCNT-LYT displayed much sharper slope of the absorbance decreasing of the cell suspension (Figure 4), that is, the faster digesting lysis of the yeast cells, demonstrating the potent antimycotic activity of LYT when loaded on PEI-functionalized CNT (Figure 2). It is worthy to note that FCNT (CNT-PEI) displayed some antimycotic activity on the tested cells of *Penicillium janthinellum*, which confirms the apparent antimycotic activity presented in Figure 2A. Nevertheless, FCNT-LYT (CNT-PEI-LYT), the loaded LYT on FCNT showed the most powerful antimycotic activity against the tested cells (Figure 4), indicating functionalization of CNT with PEI synergistically increased the specific activity of loaded LYT.

In summary, FCNT might provide a beneficial space for LYT emerging towards favorable conformational changes enabling remarkably enhanced enzyme activity, plus apparent activity of CNT-PEI. Although the detailed mechanisms for the synergetic effects of FCNT and LYT needs to be further elucidated, the potent antimycotic activities from synergistic action of antimycotic enzyme and PEI on the functionalized carbon nanotubes may find some new applications in antimycotic protections and biochemical processes.
